# Visceral Leishmaniasis Elimination Programme in India, Bangladesh, and Nepal: Reshaping the Case Finding/Case Management Strategy

**DOI:** 10.1371/journal.pntd.0000355

**Published:** 2009-01-13

**Authors:** Dinesh Mondal, Shri Prakash Singh, Narendra Kumar, Anand Joshi, Shyam Sundar, Pradeep Das, Hirve Siddhivinayak, Axel Kroeger, Marleen Boelaert

**Affiliations:** 1 International Centre for Diarrhoeal Diseases Research, Laboratory Sciences Division, Dhaka, Bangladesh; 2 Banaras Hindu University, Varanasi, India; 3 Rajendra Memorial Research Institute of Medical Sciences (ICMR), Bihar, India; 4 Institute of Medicine, Tribhuvan University, Kathmandu, Nepal; 5 Vadu Rural Health Programme, Pune, India; 6 Special Programme for Research and Training in Tropical Diseases (TDR), World Health Organization, Geneva, Switzerland; 7 Liverpool School of Tropical Medicine, Liverpool, United Kingdom; 8 Department of Public Health, Prince Leopold Institute of Tropical Medicine, Antwerp, Belgium; McGill University, Canada

## Abstract

**Objective:**

We sought to estimate visceral leishmaniasis (VL) burden in Bangladesh, India, and Nepal and document care-seeking behaviour for VL to provide baseline information for monitoring the VL elimination program and identify options for improved case finding and management.

**Design:**

A cross-sectional study using cluster sampling (clusters being villages) of 4 VL endemic districts was used in order to document all current and existing VL cases over the preceding 12 mo. Extended (in-depth) interviews were conducted in a subsample of households to explore (a) VL-related knowledge, attitudes, and practices of the population; (b) use of VL care by patients; and (c) delay between onset of symptoms, diagnosis, and start of treatment, as well as treatment interruption. Findings were discussed with national program managers and policy makers to develop improved strategies.

**Results:**

Screening for VL was done in 18,933 households (106,425 inhabitants). The estimated annual incidence of VL in the endemic districts was on average 22 times higher than the elimination target of less than one case per 10,000 inhabitants in 2015. This incidence varied widely between study sites, from 9.0 to 29.8 per 10,000 inhabitants. The percentage of newly detected cases through the household screening was high in the districts least covered by health-care services (particularly Rajshahi, Bangladesh, 49%; and to a lesser extent Vaishali in Bihar, India, 32.5%), and much lower in districts with greater availability of VL care (Muzaffarpur, India, 3.8%). On average 267 houses had to be visited, i.e., at least three to four working days per health worker, to identify a new VL (ranging from 1,432 houses in Muzaffarpur, India to only 166 houses in Rajshahi, Bangladesh). Knowledge of the disease and its vectors was good in India and Nepal but poor in Bangladesh (Rajshahi) where very little attention has been given to VL over the last decades. Although all socio-demographic indicators showed high levels of poverty, people in India preferred private medical practitioners for the treatment of VL, whereas in Nepal, and even more in Bangladesh, the public health-care sector was preferred. Delays between onset of symptoms and diagnosis as well as start of treatment was high. Reported non-adherence to treatment was particularly high in the more under-served districts and was mainly due to lack of resources.

**Discussion:**

The findings suggest that (a) house-to-house screening may be useful in highly endemic districts with a poor passive case detection system, but further evidence on case finding adapted to local conditions has to be collected; (b) strengthening the quality of the public health sector is imperative in the three countries, especially in India, with its largely unregulated private-sector provision of VL care.

## Introduction

Visceral leishmaniasis (VL) is of major public health importance in Bangladesh, India, and Nepal, affecting the poorest population groups, primarily in rural areas. More than 60% of the world's VL cases are reported from these three countries and an estimated 150 million people are at risk of VL in 109 districts [1]. The region reports 40,000 or more cases per year [1]–[4] but these official figures are likely to underestimate grossly the real burden of VL in the region [5],[6] which results in an estimated loss of 400,000 disability-adjusted life years (DALYs) annually [7].

Until recently, diagnosis and treatment of VL posed a challenge in endemic areas, because the diagnosis of VL required demonstration of the parasite in tissue aspirates of spleen, bone marrow, liver, or lymph node. These invasive diagnostic procedures were associated with a risk of severe complications such as haemorrhage and death [8]. In contrast, the rK39 dipstick test, with a sensitivity of 97% to 100% and a specificity of 86% to 92%, was found to be accurate and reliable for diagnosis when used in combination with a clinical case definition [9]–[12], and has been adopted by the VL elimination program; this initiative was launched by the three countries in 2005 with the target of reducing the annual VL incidence to less than one case per 10,000 population. For more than 60 y, VL treatment in the region consisted of injectable drugs such as the antimonial sodium stibogluconate (SAG), with decreasing cure rates. Recently, several therapeutic alternatives have become available: a recently registered oral VL drug, hexadecylphosphocholine (International Nonproprietary Name: miltefosine), the injectable paromomycin, and liposomal amphotericin B [13]–[16]. The VL elimination program has recommended the use of miltefosine until an effective combination therapy is available [17]. At the time of the study, miltefosine was available only in India, while Nepal and Bangladesh continued with antimonial treatment [18].

Further to these diagnostic and therapeutic breakthroughs, three unusual features of VL in Southeast Asia make the disease susceptible for elimination: humans are the only reservoir (anthroponotic transmission); only one vector species transmits the disease *(Phlebotomus argentipes),* whose abundance has been drastically reduced as a side effect of the World Health Organization (WHO) Malaria Eradication Programme; and the geographic distribution of the disease is limited to 109 districts, with more than 50% of VL cases occurring in the border districts of Bangladesh, India, and Nepal. Additionally, there is a strong political and administrative commitment in the three countries to eliminate VL by 2015 or earlier [2], reducing the annual VL incidence below one per ten thousand population; the governments of the three countries signed a memorandum of understanding in 2005 and are supported by WHO in this goal [18].

However, several hurdles have still to be overcome to make this target a realistic goal, and many are related to a lack of information: no sound data about VL incidence in the region exist [5]; little information about people's access to and use of diagnostic and treatment services in the public and private sector is available; and information about adherence patterns linked to a suspected lack of knowledge about the disease is limited to speculation. This information is of vital importance for designing appropriate and locally adapted VL elimination strategies. Moreover, the current approach to VL in the region is based on “passive case detection,” i.e., patients are treated if they present themselves to a health care provider. Given the low uptake of health services in this region, the overall effectiveness of the VL elimination program would be maximized were case detection organized in a more active manner, by tailoring case management approaches to local needs and addressing the issues of health services weaknesses and management constraints.

In summary, quality baseline data that will allow for comprehensive monitoring and evaluation of the progress and impact of the VL elimination program in India, Bangladesh, and Nepal are scarce. Hence, the present study was designed with the support of TDR/WHO to (a) evaluate the current caseload of VL and compare it with the elimination target, (b) describe people's health-seeking behaviour regarding diagnostic and treatment services and identify options for improvement, and (c) explore the potential for active case finding and community-based treatment strategies. This paper presents the joint analysis of data collected in four distinct study sites in three countries in order to identify similarities and differences in findings and discuss the main implications for the VL elimination initiative.

## Materials and Methods

### Study Sites

Districts with high VL endemicity in Bangladesh, India, and Nepal were identified, and, in each, subdistricts with consistently high reporting of VL cases over the preceding 3 y (reported annual incidence rates) were selected. The study districts included: Rajshahi district in Bangladesh; Vaishali and Muzaffarpur districts in Bihar State, India; and Mahottari district in South Nepal.

At the time of the study Vaishali (India) had few ongoing VL-related control activities for the last decades. Muzaffarpur (India) is known for a very active NGO (Kala-azar Medical Research Centre) doing clinical research and serving VL patients through a renowned private clinic for more than two decades. Mahottari district (Nepal) has received a United States Agency for International Development (USAID) grant in 2002–2003 for VL control, with training of health workers and village volunteers and VL awareness campaigns; there were also occasional VL-related research activities. Rajshahi district (Bangladesh) has received very little government attention so far and no external resources regarding its VL problem. No VL research has been conducted so far. When comparing the four study districts, Muzaffarpur and Mahottari were better off due to substantial support from national and international organizations; Rajshahi and Vaishali did not receive such additional resources and can be characterized as “neglected districts.”

### Sample Size for Estimating VL Case Load

For the estimation of VL case load, we used an approximation of the annual incidence rate (IR) (approximated by the number of current VL cases plus cases reported by the households that started treatment in the preceding 12 mo). We based the sample size calculation on an assumed IR of 0.10% and a desired precision of ±0.04% (95% confidence interval [CI]). Assuming a design effect of 2, the final sample size per site was 21,096.

**Figure 1:**
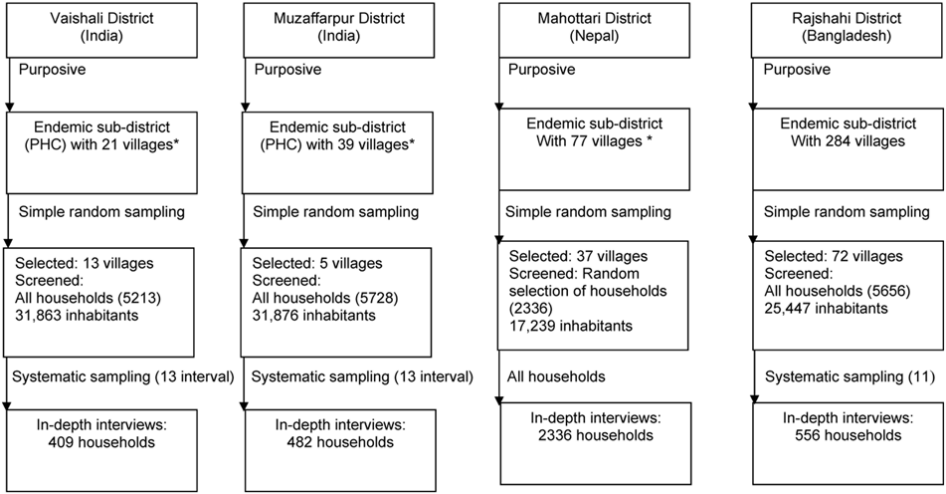
Study population and sampling. Districts and subdistricts were selected purposefully based on consistently high numbers of case reports. Villages were identified through one-stage cluster sampling (random selection of villages from a list of all villages); the screening survey covered all HHs of the selected villages. HHs for in-depth interviews were selected by systematic sampling of screening HHs. In Nepal, because of security concerns a randomly selected sample of HHs was interviewed in the study villages and the in-depth interview was applied to all of them. *The smallest administrative unit in India and Nepal is Panchyat and Village Development Committee (VDC), respectively, which frequently includes one large village and occasionally an agglomeration of smaller villages (labelled in the diagram as “village”). Primary Health Care Centres (PHC) serve a subdistrict, while the entire district is served by a district hospital.

In a subsample of households (HHs), selected by systematic sampling (Figure 1), an additional questionnaire was applied to determine people's knowledge and practices related to VL and attitudes to different health-care providers for VL treatment. Sample size calculation for this extended (in-depth) interview survey was based on the assumption that only 5% of HHs (with a required precision of ±3%, 95% CI) would consult doctors working in the public health services; the required sample size was 456 HHs. The same extended questionnaire was also applied in all households in which a past VL case was identified through the screening questionnaire.

### Sampling Procedure

The data were collected from September 2006 to March 2007 in villages randomly selected from the list of all villages in purposively selected VL-endemic subdistricts described above (Figure 1). Villages comprised roughly 100 to 1,000 houses, and all HHs were included in the sample, except for Mahottari, Nepal. There, a subsample of HHs within villages had to be taken because of security reasons (Figure 1). The study subjects were screened with the aim of identifying all past and current VL patients who were either currently on treatment or had been diagnosed within the last 1 y. HH members were screened clinically and biologically (see below) for VL. In a subsample of the houses screened and in houses with new or recent (12 mo period) VL cases an extended questionnaire was applied (“in-depth interview,” Figure 1), with the exception of Mahottari, where all households selected for the screening were also asked to participate in the extended interview.

### Survey Instruments and Organization of the Screening Survey and In-Depth Interviews

#### Questionnaires

The questionnaire of the screening survey was less than one page long and requested household identification, educational level, and occupation of the head of household. For all individual household members, the following information was collected: name, age, gender, and if they had fever during the last fortnight, if VL had been diagnosed during the preceding 12 mo and if so, in which month. Interviewees with fever were checked for an enlarged spleen and an rK39 dipstick test was done. If positive the in-depth interview was conducted and the suspected case (definition below) was referred (see below). The in-depth interview was also done with household members who had suffered from VL during the last 12 mo. This questionnaire was longer and contained sets of questions about people's knowledge and practice regarding the disease, diagnosis and treatment details, and time frames for each. The screening questionnaire generally lasted 10 min and the in-depth interview (45 questions) lasted 40 min.

#### Field staff

At each site four to six teams with two research assistants or field health workers (seven field staff at both Indian sites, eight in Nepal; eight in Bangladesh plus one experienced supervisor in each site), most of them with previous field survey experience, were recruited and trained. The study design and the questionnaire were explained in detail and the assistants did a number of test interviews, which were checked by the research team. Finally, intensive training was provided on spleen examination and conducting rK39 dipstick tests.

#### Survey procedures

Field staff conducted house-to-house visits including all HHs in the selected villages and generally applied the screening questionnaire to the heads of HHs. If they identified a HH member with fever for 2 wk or more, they performed a spleen palpation and rK39 dipstick test (KalazarDetect) according to the manufacturer's instructions (InBios International, USA). If the test was positive, the suspected VL patient was sent to the hospital together with a case report form) for further examination, particularly spleen or bone marrow aspiration. The case report form was later collected by the research team. The research assistants also conducted the in-depth interviews in a systematic sample (Figure 1) and in HHs where they identified past and present VL cases.

### Clinical Definitions and Treatment Strategy

A person with fever of more than 2 wk duration and splenomegaly was defined as a “suspect VL case”; a suspect case who was rK39 positive was defined as “probable VL case.” A “confirmed VL case” was a suspect case with a splenic or bone marrow aspiration positive for *Leishmania donovani* parasites. The analysis presented in this paper is based on all probable cases, because (a) in the three countries it is current practice to treat rK39-positive cases, (b) a number of probable cases identified by household screening in this study sought treatment in the private sector or refused any invasive diagnosis, and (c) at one site (Bangladesh) parasitological diagnosis is not done by health services at the subdistrict level. The sum of the newly detected cases by house-to-house screening and the reported cases diagnosed during the preceding 12 mo was defined as “annual VL incidence estimate” for highly endemic districts. VL deaths during the previous 12 mo were not taken into account, because cause-of-death reporting was unreliable.

### Supervision and Quality Control of Interviewers

Quality control of data collection was done through a check of all completed interviews and by a repeat of 10% of the interviews by field supervisors who asked only the core questions again. Fever history, spleen enlargement, and rK39 tests were repeated in referral centres, but not all detected patients went there (see below). Additionally, an external monitor visited the four field sites in the three countries to ensure a standardized application of the joint research protocol.

### Ethical Considerations

Before the start of the project, the protocol received clearance from the ethical committee at each study site and by the WHO Ethical Review Committee. The study participants gave written individual informed consent.

### Data Management and Analysis

As soon as data were collected from each site and checked by the supervisor, the completed questionnaires were submitted to the data processing unit at each site, where all instruments were verified manually for completeness, consistency, and manual errors. In case of any inconsistency interviewers were sent to the respective household to repeat the interview. After verification, each record was given to two data entry operators for double data entry. Data were entered in predesigned data entry program in EpiInfo 3.2.2 and then sent electronically to the central data management centre in Pune, India, where they were transferred to SPSS. The joint data analysis was done by a professional statistician at Banaras Hindu University (BHU), Varanasi, India. Through cross checks the consistency of the data was rechecked and cleaned. Finally, the data were analyzed in SPSS 10.0. Descriptive statistics as well as Chi-square test for comparison between proportions were performed. Quantitative data were expressed as average±standard deviation (SD), and categorical data as percentages with 95% CI, with online software from Dimension Research [19]. All *p*-values were two tailed and a *p*-value of ≤0.05 was taken as significant.

## Results

### Demographic and Socioeconomic Conditions (Screening Survey)

Site-specific demographic and socioeconomic conditions are shown in Table 1. A total of 18,933 households with 106,425 inhabitants were screened in the three countries (Table 2). The proportion of males was higher in Vaishali and Muzzafarpur (India), and Mahottari (Nepal) compared to Rajshahi (Bangladesh) (Table 1). The high level of poverty at all four study sites can be seen in the following indicators: (a) Very young population; 34.05% (95% CI, 33.8%–34.3%) of the study population were children under 15 y. (b) Crowded living conditions with 6.8±3.3 persons per household and an average of 2.7±2.2 rooms per house. (c) Inadequate housing, with 79.1% (95% CI, 77.8%–80.4%) living in thatched houses with mud plaster or made only with bamboo sticks. (d) High illiteracy rates; with 47.4% (95%CI, 45.8%–49.0%) of household heads were illiterate. (e) Low-paid work; 26.8% (95% CI, 25.4%–28.2%) of heads of households were landless labourers or unskilled workers. Looking at site-specific variation, there was little difference in the large proportion of children in the total population (reflecting an almost triangle-shaped population structure), but illiteracy and crowding were particularly high in Mahottari (56.4% and 7.4 persons per HH respectively), inadequate housing was frequent in Rajshahi (95.3%), and landless labourers and unskilled workers predominated in Vaishali (40%). In Muzaffarpur the indicators showed a slightly more favourable situation.

**Table 1:** Demographic and socio-economic conditions.

Indicator	Vaishali, India	Muzaffarpur, India	Mahottari, Nepal	Rajshahi, Bangladesh	*p*-Value
**Screening survey**					
Total population	31,863	31,876	17,239	25,447	—
Population aged ≤15 y, % (95% CI)	42.00 (41.44–42.52)	33.8 (33.28–34.32)	39.70 (39.00–40.46)	37.80 (37.24–38.44)	<0.0001^a^
Male sex, % (95% CI)	53.20 (52.64–53.74)	53.91 (53.36–54.46)	54.0 (53.19–54.67)	50.50 (49.93–51.15)	<0.0001^b^
Average family size±SD	6.01±3.17	5.66±2.25	7.70±5.87	4.5±1.84	<0.0001^c^
**Household survey**	*n* = 409	*n* = 482	*n* = 2336	*n* = 556	—
Age of household head, mean±SD	40.38±20.15	39.30±16.38	46.89±13.44	42.76±12.52	<0.001^d^
Household head with no education, % (95% CI)	45.23 (40.41–50.05)	41.29 (36.89–45.69)	56.38 54.37–58.39)	51.98 47.83–56.13)	<0.05^e^
HH head with unskilled/labour occupation, % (95% CI)	42.3(37.5–47.1)	38.8 (34.5–43.2)	20.21 18.6–21.8)	32.6 28.7–36.4)	<0.05^f^
Inadequate housing, % (95% CI)	85.82 (82.44–89.20)	54.36 (49.91–58.81)	76.97 (75.26–78.68)	95.32 (93.56–97.08)	<0.0001^g^
Number of rooms in house, mean (95% CI)	2.22 (2.09–2.34)	1.80 (1.70–1.90)	3.15 (3.05–3.25)	2.00 (1.89–2.10)	<0.05^g^
Persons per HH, mean±SD	6.3±3.6	6.3±2.6	7.4±3.4	4.9±2.1	<0.0001^h^

a Only Mahottari and Vaishali did not differ significantly.

b Rajshahi differs significantly from other sites.

c Each site differs from other significantly.

d Mahottari differs from all sites and Rajshahi differs from Mahottari and Muzzafarpur.

e No significant difference between Rajshahi and Mahottari as well as between Vaishali and Muzzafarpur, but Rajshahi and Mahottari differed significantly from Vaishali and Muzzafarpur.

f Vaishali and Muzzafarpur do not differ significantly.

g Each site differs significantly from other.

h Two sites of India did not differ significantly in-between them.

### Estimated VL Case Load (Screening Survey)

The screening survey (Table 2) identified 364 persons in the total study population with fever of more than 2 wk; 293 of these had a negative rK39 and were referred to the hospital for further diagnosis. Seventy-one of them (71/364, 19.5%) had splenomegaly and a positive rK39 test and were therefore classified as “VL case,” strictly speaking as “probable VL case” to be treated according to national guidelines. Additionally 166 VL cases were reported in the interviews who had been diagnosed within 12 mo preceding the interview and where the reported date of diagnosis was within that 12 mo period. Thus the average estimated annual VL incidence rate in all study sites was 22.3 per 10,000 (237/106,425; 95% CI 20.0–26.0). The proportion of cases detected through screening, out of all cases identified in the 12 mo period (i.e., reported cases within preceding 12 mo based on passive case detection [PCD], plus newly detected cases through active case finding [ACF]) was 30.0%; this proportion can be used as an indicator of the weakness of the passive case finding system across study sites.

**Table 2:** VL epidemiological findings in study areas.

Indicator	Vaishali, India	Muzaffarpur, India	Mahottari, Nepal	Rajshahi, Bangladesh	Total or Average
Population screened, *n*	31,863	31,876	17,239	25,447	106,425
Number of households screened, *n*	5,213	5,728	2,336	5,656	18,933
History of fever ≥2 wk, *n*	49	35	58	222	364
Positive rK39 test among febrile persons, *n* (%, 95% CI)	27 (55, 41–69)	4 (11, 0.89–21)	6 (10, 2.5–18)	34 (15, 10.58–20)	71 (19.51, 15.41–23)
Reported past and current VL cases during preceding 12 mo	56	65	10	35	166
Contribution of active case finding approach, % (95% CI)^a^	32.53 (22.45–42.61)	5.8 b (0.28–11.32)	37.5 (13.78–61.20)	49.28 (37.48–61.08)	29.96 (24.13–35.79)
Estimated annual incidence of VL per 10,000 population (95% CI) c	29.8 (20–32)	22.0 (17–27)	9.0 (5–13)	27.0 (21–33)	22.7 (12–30)
Houses to be visited to find one new VL case, *n* d	193	1,432	389	166	267

a (New cases/[new VL cases+past and current VL cases])×100.

b Only Muzzafarpur differed significantly from all other sites.

c ([Newly detected VL case+reported past and current VL cases]/total population)×10,000.

d Number of households screened/number of (suspected) VL cases.

There were important site-specific differences (Table 2): The highest estimated annual VL incidence rate was registered in India (Vaishali) and Bangladesh (Rajshahi), and the lowest in Nepal (Mahottari). The proportion of probable (rK39 positive) VL cases out of patients with fever was high in Vaishali, India (55.1%) but only between 10% and 15% at the other three study sites.

The proportion of cases found through ACF out of all cases identified was high in the more neglected districts such as Rajshahi, Bangladesh (where 49.3% of cases were detected only through ACF) and low in Muzaffarpur, India where case detection and treatment through NGOs has a long tradition (only 5.8% case detection through ACF); in districts with a reasonable PCD system including a reasonable level of VL awareness in the population (see below) less than half of all cases identified had been detected through ACF: 48.2% in Vaishali, India and 37.5% in Mahottari, Nepal. All rK39-positive febrile patients with enlarged spleens were parasitologically confirmed in Nepal, but only half of them in India, because many patients preferred the private sector for treatment. Parasitological diagnosis was not available in Bangladesh at the subdistrict level.

### Effort to Detect New Cases through Active Case Finding (Screening Survey)

On average 267 houses (18,933 houses/71 new VL cases) had to be visited in VL endemic villages in order to identify one probable case with prolonged fever, splenomegaly, and positive rK39 test, which corresponded to at least three to four working days of a trained interviewer or village health worker. As can be expected, the lower the disease prevalence rate, the higher the screening effort to find a new case. The number of houses to be screened was highest in Muzaffarpur, India (1,432) and lowest in Rajshahi, Bangladesh (166, Table 2).

### Knowledge about VL (In-Depth Survey)

People's knowledge about VL was poor in the neglected districts, particularly Rajshahi, Bangladesh, compared to the better served districts in India and Nepal: In India almost all interviewees (98%; 873/891 combining Muzaffarpur and Vaishali) were aware of kala-azar, the local term for VL. This was less in Bangladesh (91%; 507/556) and Nepal (82%;1,915/2,336)). Fever as the leading symptom was identified by 92% of interviewees in India, but only by 72% in Nepal and 30% in Bangladesh. Ninety-eight percent of the Indian and 97% of Nepali respondents knew that VL is curable, but only 64% in Bangladesh were aware of this (denominators as above). Likewise the knowledge about sand flies (local term in questionnaire) transmitting the disease was frequent in India (71%) and Nepal (88%) but rare in Bangladesh (21%).

### Health-Seeking Behaviour (In-Depth Survey)

**Figure 2:**
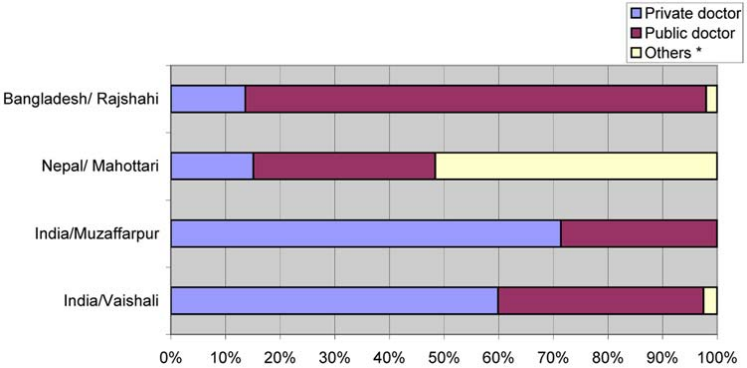
Choice of health care provider beyond community level by 113 VL patients. * Informal doctors.

Across all sites local unqualified village health workers were preferred as first-choice health care providers. This choice was associated with their excellent accessibility (on average 15 min travel time). Choosing health care beyond the community, the Indian respondents preferred private providers over governmental ones while in Bangladesh and Nepal public services were preferred over the private ones (Figure 2). The choice between private or public health care professionals was not dependent on travel times and transport costs to private and public practitioners, because these were similar in the study sites or, in Bangladesh, even longer/more expensive to reach the preferred government doctors. Respondents in India would use for the treatment of VL mainly the private sector (50%) and less the public sector (30%), while in Nepal and particularly in Bangladesh the pattern was the reverse: mainly use of public sector (Nepal 45%; Bangladesh 52%) and less of private practitioners (11% and 13%, respectively). Additionally, in India people resorted to local unqualified village health workers for VL treatment (12%), in Nepal to indigenous healers (23%), and in Bangladesh to local chemists (28%). Main reasons for choice of health care provider for VL treatment were: geographical accessibility for village health workers, indigenous healers, and local chemists; but for selecting between private and public sector the most frequently mentioned factors were “faith” (belief that VL can be treated adequately) and “good interpersonal communication.”

The selection pattern between private and public health sector for treatment of kala-azar was roughly confirmed by interview answers of 113 current and past (<12 mo) VL patients in the in-depth study (Figure 2; not all patients identified answered): In India 80% (53/70) had used the private sector and 20% the public sector. In Bangladesh the use of public sector was 81.2% (28/35). Of the eight patients interviewed in Nepal, six (75.0%) went to the public sector and two (25%) went to local chemists.

### Delay in Diagnosis and Treatment (In-Depth Study of VL Patients)

**Figure 3:**
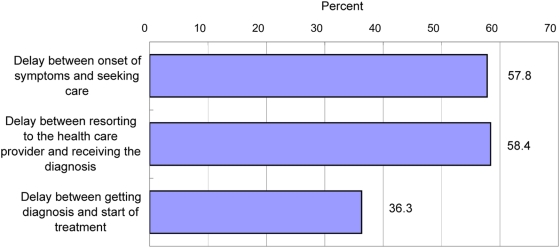
Reported treatment delay of more than 2 wk from onset of symptoms to diagnosis and start of treatment as a proportion of 113 VL patients in India, Bangladesh, and Nepal.

Delays of more than 2 wk (Figure 3) between onset of symptoms (“feeling ill”) and seeking care was frequent (57.8% of the 113 VL patients who responded) with the highest proportion in Rajshahi, Bangladesh (65.7%). The delay between resorting to the health care provider and receiving the diagnosis was 1 wk or less in most cases (58.4%), but particularly long in Vaishali, India, because of the outsourcing of diagnostic services from public hospitals and health centres to private laboratories (58.8% with more than 4 wk delay). There was significant delay between diagnosis and start of treatment by more than 2 wk particularly in the neglected districts of Vaishali and Rajshahi.

### Treatment Interruption and Reasons (In-Depth Interview)

Of 113 VL patients, 22.8% interviewed reported having interrupted the treatment at one stage. In most cases (85.7%) the reported interruption was 2 wk or less; this was particularly the case in Muzaffarpur, India and Mahottari, Nepal; however, in Rajshahi, Bangladesh half of VL patients and in Vaishali, India, one-third of patients had interrupted their treatment for more than 3 wk. Main reported reasons for treatment interruption were lack of money for treatment (68.7%) and side effects (15.7%).

## Discussion

### Limitations of the Study

The four study sites in three countries illustrate the variety of epidemiological, ecological, social, and health services conditions that shape VL transmission in the region. The information was collected in highly endemic districts and cannot be extrapolated to other areas. Furthermore, the large variation between sites enriches the interpretation but also highlights the need for more detailed site-specific analyses (unpublished data).

### Explanatory Factors for Variation of Estimated VL Incidence across Sites

Possible explanatory factors for the variation of VL incidence (estimated VL incidence three times higher in Rajshahi, Bangladesh compared to Mahottari, Nepal) include:

- Serious attempts at earlier case detection and treatment have been made in Muzaffarpur, India and to a lesser extent in Mahottari, Nepal. (PCD was considerably more successful in Muzaffarpur with 94.2% of cases detected compared to Rajshahi, Bangladesh with only 50.7% of cases detected through PCD; delay between feeling sick and getting treatment was particularly low in Muzaffarpur and high in Rajshahi and Vaishali, India.)
- People's exposure to VL-related health messages, particularly in Muzaffarpur and less in Mahottari as compared to Rajshahi has increased awareness about the disease and the vectors (for example, awareness of fever as leading symptom and mosquitoes as transmitting agents was high in Muzaffarpur and low in Rajshahi) and—together with a better availability of treatment facilities and drugs—has possibly contributed to faster care seeking and better adherence to treatment schedules.
- Vector control, although still insufficient and of questionable quality in India and Nepal, seems to impact on the vector population compared to Bangladesh where vector control has largely been abandoned during the preceding decades [20],[21].

### Recognition of Study Results by the Regional Technical Advisory Group

The study has provided important evidence to be considered in designing the case detection/case management components of the kala-azar (VL) elimination strategy. These points were discussed in 2007 and 2008 with RTAG (Regional Technical Advisory Group for the Kala-azar Elimination programme), which includes key stakeholders from Ministries of Health, academia, WHO/SEARO, and donors (World Bank) in the three countries, and it advises policy makers on the technical aspects of the elimination strategy. The following findings were identified as being particularly relevant and leading to action:

- The actual estimated annual VL incidence in the highly endemic study districts is roughly 22 times higher than the elimination target for 2015 (less than one per 10,000); although not representative for all endemic areas, repeated progress monitoring through screening surveys in sentinel sites (“indicator areas”) of highly endemic districts using the simple data collection tool of this study are recommended for monitoring operational progress and health impact. The cost and feasibility of such a monitoring system through the public health sector should be assessed, and further districts should be included to have a broader basis of baseline data to allow for monitoring of progress.
- Active case finding in high-endemicity districts/subdistricts through household screening for fever patients with subsequent spleen palpation and rK39 testing and treatment seems to be feasible (on average 272 households have to be visited by a village health worker, which means between three and four working days) but should be restricted to districts where PCD is weak and thus the yield of ACF detected cases can be expected to be high; further studies are recommended about different models of ACF (house-to-house screening, mobile clinics, incentives for detecting a new case, index case–triggered screening) and about their operational costs (cost per case detected) compared to improved PCD. Active case finding could help not only to shorten treatment delay by preventing “healer shopping” with an indiscriminate and expensive use of a large variety of formal and non-formal health care providers [22]–[24], but also to keep newly detected patients in the public sector; however, evidence has to be collected to test this assumption.
- The easily accessible unqualified village health workers could be (better) trained to spread important health messages and eventually participate in the early provisional VL diagnosis by alerting people with fever and facilitating the access to rapid diagnosis.
- Outsourcing of VL diagnostic services in some parts of India including the two study districts should be re-assessed, because it contributes to a delay in diagnosis (as shown for Vaishali district) and treatment.
- Special attention should be paid to the role of the private health sector, particularly in India, because our study and others [25],[26] have documented high utilization of private medical services with questionable VL treatment practices. Likewise, the extensive use of local chemists in Nepal (Figure 2) needs more attention. With the advent of oral drugs the tendency for uncontrolled and inadequate drug use may be increased, so a realistic policy response is urgently needed for both India (private sector regulations) and Nepal (monitoring of private pharmacies). VL treatment is free of charge in the public hospitals of Bangladesh, India, and Nepal. Nevertheless, in all three countries a realistic assessment of health services performance and options for improvement should be undertaken and alternative case management strategies—such as DOT (directly observed therapy), including monitoring of side effects and patient satisfaction—should be developed and validated.
- “Information, education, communication” (IEC) campaigns, particularly in Bangladesh, would be useful for improving people's knowledge about the disease and its prevention and treatment, but costs and expected outcomes for such campaigns should be established.

In view of the many barriers for reaching the elimination goal, a massive effort in terms of up-scaling human resources, supplies, logistics, and monitoring activities will be required. This study has provided important baseline information necessary for monitoring progress. Additional research in a second phase of studies, as outlined above and to be designed jointly by the research teams and VL country programme managers, will contribute to adapting the elimination strategy to needs and identifying implementation plans with a focus on cost effectiveness, safety, and final outcomes of VL case finding and treatment through the primary health care system with the back-up of hospitals. The RTAC highlighted the need for continued interaction between researchers, national program managers, and political decision makers. The main challenge for the VL elimination initiative is to increase access to VL care in these underserved areas; exploring active case finding also seems worthwhile. Meanwhile, the main measure to attract patients to the public health system would be to remove any financial barriers (fees for consulting, for investigations, for drugs, etc.) and to increase quality of care. Additionally, regulation of the private sector should be attempted to improve the quality of VL care.
